# Fear Inhibition in High Trait Anxiety

**DOI:** 10.1371/journal.pone.0086462

**Published:** 2014-01-16

**Authors:** Merel Kindt, Marieke Soeter

**Affiliations:** 1 Department of Clinical Psychology, University of Amsterdam, Amsterdam, The Netherlands; 2 Research Priority Program Brain and Cognition, Cognitive Science Center Amsterdam, University of Amsterdam, Amsterdam, The Netherlands; Universidad de Granada, Spain

## Abstract

Trait anxiety is recognized as an individual risk factor for the development of anxiety disorders but the neurobiological mechanisms remain unknown. Here we test whether trait anxiety is associated with impaired fear inhibition utilizing the AX+/BX- conditional discrimination procedure that allows for the independent evaluation of startle fear potentiation and inhibition of fear [1]. Sixty undergraduate students participated in the study - High Trait Anxious: n = 28 and Low Trait Anxious: n = 32. We replicated earlier findings that a transfer of conditioned inhibition for startle responses requires contingency awareness. However, contrary to the fear inhibition hypothesis, our data suggest that high trait anxious individuals show a normal fear inhibition of conditioned startle responding. Only at the cognitive level the high trait anxious individuals showed evidence for impaired inhibitory learning of the threat cue. Together with other findings where impaired fear inhibition was only observed in those PTSD patients who were either high on hyperarousal symptoms [2] or with current anxiety symptoms [3], we question whether impaired fear inhibition is a biomarker for the development of anxiety disorders.

## Introduction

Current etiological models of anxiety disorders emphasize the importance of individual risk factors such as neuroticism or trait anxiety. However, most evidence comes from correlational studies in which personality traits are related to psychopathology at later points in time. Such correlational studies are in fact non-informative given that neuroticism or trait anxiety largely overlaps with symptoms of anxiety disorders [4]. Pavlovian fear conditioning is well suited to investigate the *neurobiological mechanisms* by which personality traits may contribute to the development of pathological fears. In human fear conditioning studies an initial neutral or ambiguous stimulus (i.e., the conditioned stimulus or CS; e.g., a picture) acquires the capacity to elicit fear responses after being followed by an intrinsically aversive stimulus (i.e., the unconditioned stimulus or US; e.g., an electric shock). Conditioned fear responding is indexed by larger fear responses elicited by a conditioned stimulus (i.e., CS+) relative to a safety signal (i.e., CS-) that is never paired with the shock. Objective measurements such as potentiation of the eye blink startle reflex or subjective measurements such as shock expectancy ratings are typically used to assess conditioned responding. Animal and human research has demonstrated that the fear potentiated startle reflex (i.e., FPS) is a reliable and specific index of fear, directly connected with and modulated by the amygdala [5], [6]. The FPS is considered to be an excellent tool for translational research because it is a well-characterized and neurobiological measure of fear that can be used across mammalian species [7].

Previous differential fear-conditioning studies (CS+ *vs*. CS-) in individuals at risk for anxiety do not show elevated fear responding to the conditioned threat stimulus (CS+), but show elevated fear responding to the safety cue - the stimulus that does not signal threat during conditioning (CS-) or the stimulus that no longer signals threat during extinction [8]–[12]. While these findings seem to be in line with the hypothesis of impaired fear inhibition as a biomarker for the development of anxiety disorders [13], the traditional fear-conditioning paradigm falls short in distinguishing between excitatory and inhibitory learning. Extinction learning does not erase the original fear memory but involves the formation of a new inhibitory stimulus association (i.e., CS - no shock) that competes with the original fear memory (i.e., CS-US) [14], [15]. The competition between the original excitatory fear association and the newly formed inhibitory memory trace determines the behavioral outcome of extinction learning [16]. Hence, impaired extinction learning is not necessarily due to deficits in inhibitory learning but may also be the result of enhanced excitatory learning during acquisition [14], [17]. In a similar vein, enhanced responding to the safety cue (CS-) during fear acquisition may also be explained by enhanced responding to the CS+ (i.e., excitatory learning), which in turn promotes generalization from the CS+ to the CS- [18]–[20].

Here we test whether trait anxiety is associated with impaired fear inhibition utilizing the AX+/BX- conditional discrimination paradigm that allows for the independent evaluation of fear excitation and fear inhibition [1], [16]. Healthy volunteers divided into a high and low trait anxiety group were presented with one set of colored figures paired with the delivery of an electric shock to the wrist (i.e., AX+) and a different series of colored figures presented without aversive shock (i.e., BX-). Our procedure marginally differed from the original version developed by Jovanovic et al. [1]: instead of colored lights we presented colored figures as AX+ or BX- stimuli and instead of an aversive air blast to the throat we delivered an electric shock to the wrist as US. In this AX+/BX- conditional discrimination paradigm, responses to the third stimulus X are conditional upon the presence of either A or B. During acquisition stimulus A becomes excitatory as participants learn that the presentation of A and X together predicts the unconditioned stimulus (US). Stimulus B becomes inhibitory given that the presentation of B with X predicts safety from the US. A subsequent presentation of A and B together should result in reduced startle fear potentiation to stimulus A because stimulus B transfers its inhibitory property to stimulus A [1], [21], [22]. Evidence for transfer of fear inhibition would thus appear from lower conditioned startle responding to AB compared to a novel compound stimulus AC [23]. Impaired fear inhibition has already been reported in veterans with PTSD [3] and in civilians with high levels of urban trauma [2]. As impaired fear inhibition may also be the consequence of an anxiety disorder rather than a vulnerability factor for developing the disorder, an interesting question is whether impaired fear inhibition will also be observed in individuals at risk for anxiety disorders.

In previous studies on inhibition of the fear potentiated startle response, about one third of the participants did not learn to discriminate between the threat and safety cues during the fear-conditioning phase and did not show transfer of inhibition [1], [21]. As awareness of the contingencies appeared to be necessary for fear inhibition, Jovanovic et al. excluded the unaware participants from further analyses [1], [21]. This finding clearly deviates from observations where conditioning of the FPS is independent from participants' threat expectations [24]–[27]. We therefore also test whether explicit knowledge of the threat and safety cues during learning is required for the transfer of fear inhibition. To address this question, the unaware participants will not be excluded, but the online expectancy ratings will be included as covariate and between-subjects factor in the statistical analyses of fear inhibition.

## Materials and Methods

### Participants

Sixty undergraduate students from the University of Amsterdam ranging in the age of 18 to 55 years (mean ± SD age, 22.9±6.6 years) participated in the study. Participants were subdivided into two groups on the basis of the median split score of the trait anxiety scale of the State-Trait-Anxiety Inventory [28] - *low anxiety* (n = 32 - mean STAI-T ± SD, 29.6±3.5) and *high anxiety* (n = 28 - mean STAI-T ± SD, 47.0±8.0). Participants were assessed to be free from any auditory or visual impairment. Participants received either partial course credits or were paid a small amount for their participation in the experiment. Our ethical committee of the University of Amsterdam approved the study and written informed consent was obtained from all participants.

### Apparatus and Measurements

#### Stimuli

**Figure 1:**
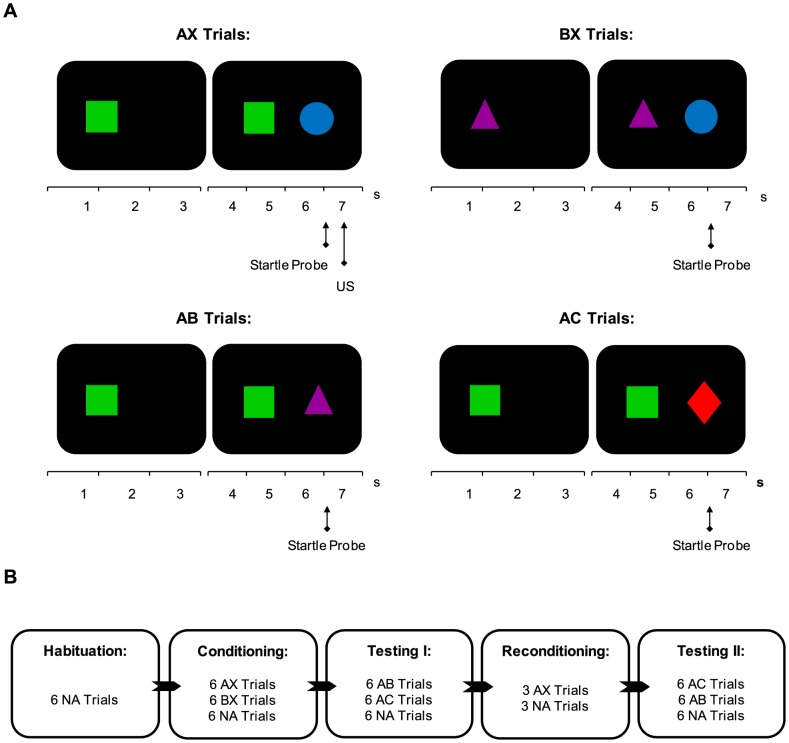
Experimental design. **A** Structure of the AX - BX - AB and AC trials. **B** Schematic of the different phases of the experiment.

Conditioned stimuli (CS) consisted of four colored figures (see Fig. 1a) with a maximum cross section of 10.75 cm. Stimuli were presented left or right from the middle of a black screen on a 19-in computer monitor with a fixed location for each stimulus. An electric shock with duration of 2 ms delivered to the wrist of the non-preferred hand served as US. Delivery of the US was controlled by a Digitimer DS7A constant current stimulator (Hertfordshire - UK) via a pair of Ag electrodes of 20 by 25 mm with a fixed inter-electrode mid-distance of 45 mm. A conductive gel (Signa - Parker) was applied between the electrodes and the skin.

#### US Expectancy Ratings

Expectations of the US were measured using a pointer on a continuous scale consisting of 11-points labeled from “certainly no electric shock” through “uncertain” to “certainly an electric shock”. Participant's ratings were presented at the bottom of the computer screen in order to encourage participants to focus their attention to the CSs. Participants were required to rate the expectancy of an electric shock during the presentation of each figure by shifting the pointer on the scale and push the left mouse button.

#### Fear Potentiated Startle

Conditioned fear responding was measured as potentiation of the eye blink startle reflex to a loud noise by electromyography (EMG) of the right orbicularis oculi muscle. Two 2.5 mm Ag-AgCl electrodes filled with electrolyte gel were positioned 1 cm under the pupil and 1 cm below the lateral canthus. A ground reference was placed approximately 5 cm below the orbicularis oculi pars orbitalis on an electrically neutral site. A loud noise (40 ms - 104 dB) was administered during each CS presentation and during intertrial intervals (i.e., NA - noise alone trials). All acoustic stimuli were delivered binaurally through headphones (Model MD-4600 - Compact Disc Digital Audio - Monacor). Eye blink EMG activity was measured using a bundled pair of electrodes wires connected to a front-end amplifier with an input resistance of 10 MΩ and a bandwidth of DC-1500 Hz: the amplifier was designed around a Burr Brown INA101 instrumentation amplifier and ISO103 isolation stage by B. Molenkamp - UvA. To remove unwanted interference a notch filter was set at 50 Hz. Raw EMG signals were integrated in the amplifier: integration was handled by a true-RMS converter with a time constant of 25 ms. Integrated EMG signals were sampled at 500 Hz and used for data analysis. Integrated peak amplitudes were determined by taking the baseline − 50 ms before probe onset - to peak difference within the 20–200 ms following probe onset and were recorded in microvolts.

#### Subjective Assessments

Trait anxiety was assessed with the State and Trait Anxiety Inventory [28].

### Experimental Procedure

Throughout the experiment participants sat in front of a computer monitor at a distance of 70 cm in a sound-attenuated room. After written informed consent was obtained the EMG and shock electrodes were attached and the intensity of the US was determined. Starting at an intensity of 1 mA the level of a 2-milliseconds aversive electric shock delivered to the wrist of the non-preferred hand was gradually increased in steps of 3 mA. Intensity of shock was individually set at a level defined by the participant as “uncomfortable - but not painful”. Next the participants were informed regarding the US expectancy measures. They were instructed that they should learn to predict whether an electric shock would occur or not on the basis of the colored figures. Participants were required to rate the shock expectancy on all of the trials.

Testing started with a 1-min acclimation period consisting of 70 dB broadband noise - which continued throughout the session as background noise - followed by a habituation phase consisting of six baseline startle probes (i.e., noise alone (NA) trials) to reduce initial startle reactivity. Conditioning included six trials in which stimuli A and X were paired with the US as well as six trials in which stimuli B and X were not paired with the US and six NA trials [1] (see Fig. 1b). Order of trial was quasi-random with the restriction that no more than two consecutive trials were of the same type. Within a trial the two stimuli were presented serially - order of the two stimuli was alternated randomly across trials. In the AX trials the first stimulus was presented for 7 s. After 3 s the second stimulus was presented. The startle probe was presented at the end of 6 s and was followed by the electric shock 500 ms later (see Fig. 1a). In the BX trials - as well as in the AB and AC test trials - no electric shock was presented.

Testing phases consisted of two blocks. Each block included six presentations of either AB or AC and six NA trials (see Fig. 1b). Order of the two blocks was counterbalanced across participants - (1) order AB *vs.* AC: high trait anxiety n = 15 and low trait anxiety n = 17 - (2) order AC *vs.* AB: high trait anxiety n = 13 and low trait anxiety n = 15. In between the blocks there was a brief reconditioning phase in which the AX trials were presented three more times with the US to maintain fear potentiation to AX. In all phases of the experiment the inter-trial intervals were randomized - ranging from 9 to 22 s. Upon completion of the experiment participants completed the STAI.

### Statistical Analysis

Expectancy ratings and startle responses were analyzed by means of mixed factor analyses of variance with trial - AX - BX - AB - AC - as within-subject factor and group - high trait anxiety *vs.* low trait anxiety - as between-subject factor. Startle potentiation was calculated by subtracting the baseline startle amplitude (i.e., NA) from the startle amplitude during the corresponding test trial. We controlled for the variable nature of the startle responding by averaging two trials of each of the trial types. For the US expectancy ratings only one trial of each of the trial types was analyzed given that the effect of learning can easily be observed on the cognitive level of fear. Results of fear acquisition were captured by comparing the last (two) trial(s) of AX and BX. For the transfer of fear inhibition we compared the first (two) trial(s) of AB and AC. Given that Jovanovic et al. [1], [2] also assessed fear inhibition by comparing conditioned responding to the last (two) trial(s) of AX with the first (two) trial(s) of AB, we also present these analyses such that our results can be compared with their previous studies. Outliers were replaced by mean values across trial types −>3 SD − 1.51% of the US expectancy ratings − 2.43% of the startle responses. Missing startle responses − 0.6% of the trials - were excluded from the analyses.

## Results

**Figure 2:**
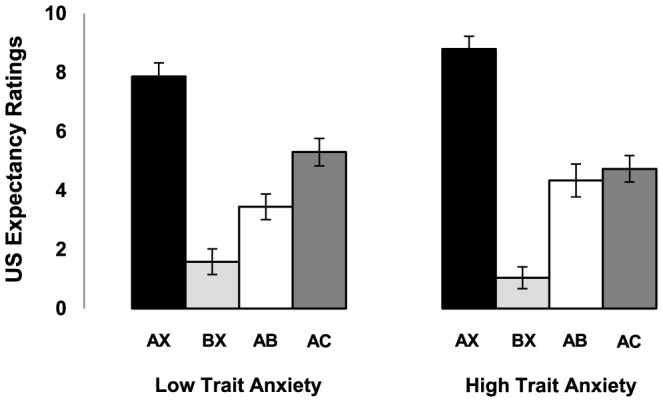
US expectancy ratings. Expectancy of the unconditioned stimulus to the last AX and BX trial as well as the first AB and AC trial for the *low trait anxiety* and *high trait anxiety* group. Error bars represent SEM.

High trait anxious and low trait anxious participants differed significantly in their trait anxiety [*t*_58_ = 11.18, *P*<0.001, two-tailed]. We further observed no differences in selected shock intensity between the *high trait anxiety* and *low trait anxiety* group [*t*_58_<1]. Both groups showed similar fear learning by a significant differential increase in US expectancy from the first acquisition trial to the last trial of acquisition [AX *vs.* BX - stimulus x trial, *F*_1,58_ = 256.53, *P*<0.001, η^2^ = .82; stimulus x trial x group, *F*_1,58_<1.22]. Comparing the expectancy ratings to AB with the ratings to a novel compound AC revealed however a significant difference between groups [stimulus x group, *F*_1,58_ = 4.24, *P*<0.05, η^2^ = .07; Fig. 2]. Whereas the LTA group showed a significant fear inhibition effect [AB *vs.* AC - *t*_31_ = 3.68, *P* = 0.001, two-tailed] we observed no fear inhibition in the HTA group [AB *vs.* AC - *t*_27_<1; Fig. 2]. An additional linear regression analysis with fear inhibition as dependent variable - i.e., calculated by subtracting the expectancy ratings on the first AB trial from the expectancy ratings on the first AC trial - and trait anxiety scores entered as the independent variable indeed confirmed this finding: higher trait anxiety scores were correlated with less fear inhibition when comparing AB to a novel compound AC [R^2^ = 0.08, *t*_58_ = 2.25, *P*<0.05].

**Figure 3:**
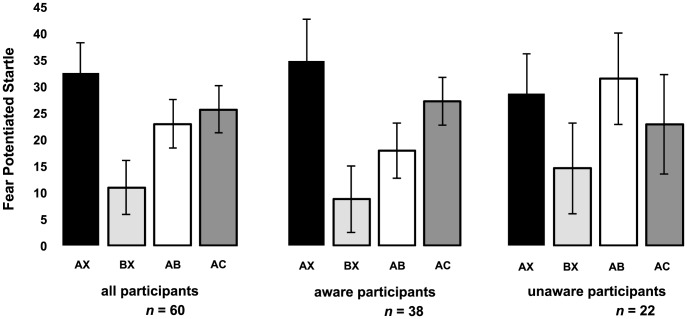
Fear potentiated startle responding. Mean startle potentiation to the last two AX and BX trials and the first two AB and AC trials for *all* the participants as well as the *aware* and *unaware* participants following the Jovanovic et al. criterion [1].

Analysis of the *startle reflex* to noise alone revealed similar levels of startle responding during acquisition as well as during the testing phases in the HTA and LTA groups [trial x group, *F*s_5,52_<1.31]. We further observed similar fear learning in both groups as is indicated by a significant differential increase in startle fear responding from the first two acquisition trials to the last two trials of acquisition [AX *vs.* BX - stimulus x trial, *F*_1,58_ = 8.47, *P*<0.01, η^2^ = .13; stimulus x trial x group, *F*_1,58_<1.19]. An inhibition effect was however absent in both groups [AB *vs.* AC - stimulus, *F*_1,58_<1; Fig. 3]. Note that Jovanovic et al. [1], [21] only observed startle fear inhibition in those participants that were aware of the experimental contingencies according to their scoring on the US expectancy ratings. Utilizing the criterion for awareness described by Jovanovic et al. [1] we carried out follow-up analyses with contingency awareness as between-subject factor. Participants had to have two consecutive correct responses to each of the two types of training trials. A second criterion was to correctly label the last training trial of each type. We operationally defined correct responses to AX+ trials as expectancy scores of 7 or higher when presented with the A figure as well as during an X figure when it followed A. Correct response to BX- trials were expectancy scores of 3 or lower when presented with the B figure as well as during an X figure when it followed B. Subjects who did not meet both criteria were defined as unaware. These analyses indeed revealed a significant difference in fear inhibition between the aware and unaware participants [AB *vs.* AC - stimulus x group, *F*_1,58_ = 5.38, *P*<0.05, η^2^ = .09]. Whereas the aware participants showed a significant fear inhibition effect [AB *vs.* AC - stimulus, *F*_1,36_ = 4.47, *P*<0.05, η^2^ = .11], the fear inhibition effect was absent in the unaware participants [AB *vs.* AC - stimulus, *F*_1,21_<1.46]. Given that these criteria for contingency awareness are somewhat arbitrary, we included degree of contingency awareness as a covariate factor in further analyses. Degree of contingency learning was calculated by subtracting the differential expectancy ratings (i.e., AX *vs.* BX) on the first trial of fear acquisition from the differential expectancy ratings on the last acquisition trial (i.e., trial 6). Participants in the HTA and LTA group did not differ on the degree of contingency learning [*t*_58_<1.27]. We also observed a significant fear inhibition effect with contingency awareness as covariate [AB *vs.* AC - stimulus, *F*_1,57_ = 8.43, *P*<0.01, η^2^ = .13]. In sum these data indicate that the expectancy ratings during learning strongly affected the later startle fear inhibition (see also Fig. 3). Note however that the startle differentiation during fear learning [AX *vs.* BX] was not related to fear inhibition of startle potentiation [AB *vs.* AC - *r*_spearman_ = 0.03, *P* = 0.85]. But most pertinent to our hypotheses - with or without the degree of contingency awareness as a covariate factor - we observed no difference in fear inhibition between the HTA and LTA group [AB *vs.* AC - stimulus x group, *F*s_1,58_<1]. Also two-step hierarchical regression analyses with transfer of inhibition as dependent variable - i.e., calculated by subtracting the mean startle fear responding on the first two AB trials from the mean startle fear responding on the first two AC trials - and degree of contingency awareness and trait anxiety entered in step one and step two respectively failed to detect any relationship between fear inhibition and trait anxiety for the startle fear responding [AB *vs.* AC - *P*>0.05].

### Additional analyses - AX vs. AB

Jovanovic et al. [1], [2] also assessed fear inhibition by comparing conditioned responding to AX with AB. Accordingly we carried out similar analyses on the US expectancy ratings and startle responses. When comparing the expectancy ratings to AX with the ratings to AB we observed a significant but similar decrement in US expectancy in the HTA and LTA group [stimulus, *F*_1,58_ = 94.25, *P*<0.001, η^2^ = .62; stimulus x group, *F*_1,58_<1; Fig. 2]. Yet for the startle responding we again did not reveal any differences [AX *vs.* AB - stimulus, *F*_1,58_<2.80; Fig. 3]. However follow-up analyses with contingency awareness as a between-subjects factor also revealed significant differences in conditioned startle responding between the aware and unaware participants [AX *vs.* AB - stimulus x group, *F*_1,58_ = 4.12, *P*<0.05, η^2^ = .07]. Whereas the aware participants showed significantly less conditioned startle responding to AB compared to AX [stimulus, *F*_1,36_ = 4.80, *P*<0.05, η^2^ = .12], this effect was absent in the unaware group [AX *vs.* AB - stimulus, *F*_1,21_<1]. Also when the degree of contingency awareness was included as a covariate factor we observed a significant reduction in startle responding [AX *vs.* AB - stimulus, *F*_1,57_ = 5.42, *P*<0.05, η^2^ = .09]. Together these findings correspond to those of previous studies on fear inhibition [1], [2]. We further observed no differences in conditioned startle responding between the HTA and LTA group [AX *vs.* AB - stimulus x group, *F*s_1,57_<1].

## Discussion

Here we replicated the original findings of Jovanovic et al. [1] by demonstrating (1) transfer of conditioned inhibition in a human fear-potentiated startle paradigm and (2) that fear inhibition is dependent on explicit knowledge of the threat and safety signals. We further show that (3) high trait anxiety was not associated with impaired fear inhibition as evident from startle responding - but we found impaired fear inhibition at the cognitive level (AB *vs.* AC). Thus, while the high trait anxious individuals seemed not to trust the safety cues, the physiological expression of fear inhibition was not affected by trait anxiety. While the sample sizes were not too small to unveil a difference between groups (i.e., post hoc power for the crucial test of fear inhibition with α = 0.05 and Cohen's d = 0.50 showed a power of 0.68), the absence of a difference in startle potentiation between the high and low trait anxiety groups may however still be due to a type II error.

Even though the fear potentiated startle and the shock expectancy ratings do not show a similar pattern on the test of fear inhibition, explicit knowledge of the threat and safety cues seemed to be required for a later transfer of startle fear inhibition. Our findings - among previous studies showing that contingency awareness is required for fear inhibition [1], [21] - question whether the AX+/BX- paradigm taps into the neural underpinnings of fear inhibition. It may even be suggested that fear inhibition is rather consequential to a reduced expectancy of the aversive shock, which in turn prevents the expression of fear potentiated startle responses. While startle potentiation may operate relatively independent from contingency awareness in traditional fear conditioning paradigms [24]–[27], contingency awareness seems also required for more complex fear conditioning procedures like trace conditioning in which a temporal gap is involved between the CS and US presentation [29]. Research into the neural underpinnings of fear conditioning has demonstrated that both contingency awareness and conditioned fear responding in complex learning tasks are dependent on intact hippocampal functioning [30]–[33]. A developing literature shows that the hippocampus is also involved in the contextual encoding and context-dependent retrieval of fear extinction [34]. Although it is appealing to suggest that the underlying processes of fear inhibition and fear extinction overlap, the neural underpinnings of fear inhibition are largely unknown.

Our suggestion that the absence of a difference in startle fear inhibition between the high trait anxiety and low trait anxiety group may also be due to a type II error seems to be corroborated by other findings where impaired fear inhibition in PTSD patients was reported [2], [3]. An alternative explanation for this discrepancy may however be sought in some procedural differences between our study and those by Jovanovic et al. [2], [3], with the most notable difference being the analysis of fear inhibition. Firstly, during fear conditioning no difference in startle potentiation was observed between the threat stimulus AX+ and the safety stimulus BX- in patients with PTSD. Lack of transfer of fear inhibition (i.e., increased startle potentiation to AB) may also be due to a generalization of excitatory learning to the safety cue (from AX+ to BX-). Even though the difference between AX+/BX- startle potentiation is not related to the difference between AB/AC startle potentiation, the absence of discriminative fear learning makes the reported lack of fear inhibition difficult to interpret. Thus, the impaired fear inhibition in patients with PTSD may be secondary to the lack of fear discrimination, as was also suggested by the authors [2]. Secondly, fear inhibition was actually assessed with an invalid test of fear inhibition by comparing AX versus AB as opposed to the critical comparison of AB versus AC [1], [2], [23]. However, the authors were critical on the AB/AC comparison given that the C of the AC compound is completely novel when first presented at test, whereas the B had already been presented before it appeared in the new compound AB [3]. But even considering the less valid test of inhibition (i.e., AX vs. AB), a noticeable finding is that impaired fear inhibition was only observed in those PTSD patients who were either high on hyperarousal symptoms [2] or with current anxiety symptoms [3]. Individuals with a history of PTSD - but with low current symptoms - responded similarly to healthy controls and showed intact fear inhibition. These findings undermine the hypothesis of impaired fear inhibition as biomarker for the development of anxiety disorders. They rather suggest that impaired fear inhibition may be consequential to high anxiety states.

To summarize, our data suggest that individuals at risk for anxiety have only impaired fear inhibition at the cognitive level. Together with the finding that explicit knowledge of the threat and safety cues is a necessary condition for later inhibition of startle fear responses, we conclude that there is no strong evidence for the hypothesis of impaired fear inhibition as a biomarker for the development of anxiety disorders.
